# Alterations in Gut Microbiota Are Correlated With Serum Metabolites in Patients With Insomnia Disorder

**DOI:** 10.3389/fcimb.2022.722662

**Published:** 2022-02-17

**Authors:** Jing Zhou, Xiaoling Wu, Zhonglin Li, Zhi Zou, Shewei Dou, Gang Li, Fengshan Yan, Bairu Chen, Yongli Li

**Affiliations:** ^1^ Department of Health Management, Henan Key Laboratory of Chronic Disease Health Management, Henan Provincial People’s Hospital, Henan University People’s Hospital, Zhengzhou, China; ^2^ Department of Nuclear Medicine, Henan Key Laboratory of Chronic Disease Health Management, Henan Provincial People’s Hospital, Zhengzhou, China; ^3^ Department of Radiology, Henan Provincial People’s Hospital, Zhengzhou, China; ^4^ Department of Medical Imaging, Henan Provincial People’s Hospital, Zhengzhou, China; ^5^ Department of Neurology, Henan Provincial People’s Hospital, Zhengzhou, China

**Keywords:** gut microbiota, insomnia disorder, metabolic profile, serum metabolites, microbiota-brain communication

## Abstract

This study aimed to investigate insomnia-related alterations in gut microbiota and their association with serum metabolites. A total of 24 patients with insomnia disorder and 22 healthy controls were recruited. The fecal and serum samples were collected. The 16s rRNA sequencing and bioinformatics analysis were conducted to explore insomnia-related changes in the diversity, structure, and composition of the gut microbiota. UPLC-MS was performed to identify insomnia-related serum metabolites. Spearman correlation analysis was used to investigate the correlations between insomnia-related gut bacteria and the serum metabolites. Despite the nonsignificant changes in the diversity and structure of gut microbiota, insomnia disorder patients had significantly decreased family *Bacteroidaceae*, family *Ruminococcaceae*, and genus *Bacteroides*, along with significantly increased family *Prevotellaceae* and genus *Prevotella*, compared with healthy controls. Genus *Gemmiger* and genus *Fusicatenibacter* were dominant in patients with insomnia disorder, whereas genus *Coprococcus*, genus *Oscillibacter*, genus *Clostridium XI*, and family *Peptostreptococcaceae* were dominant in healthy controls. The UPLC-MS analysis identified 97 significantly decreased metabolites and 74 significantly increased metabolites in the serum samples of patients with insomnia disorder, compared with those of healthy controls. KEGG enrichment analysis revealed 1 significantly upregulated metabolic pathway and 16 downregulated metabolic pathways in patients with insomnia disorder. Furthermore, Spearman correlation analysis unveiled significant correlations among the altered bacteria genus and serum metabolites. Patients with insomnia disorder have differential gut microbiota and serum metabolic profiles compared with healthy controls. The alterations in gut microbiota were correlated with specific serum metabolites, suggesting that some serum metabolites might mediate gut microbiota-brain communication in the pathogenesis of insomnia disorder.

## Introduction

Insomnia is a sleeping disorder characterized by difficulty in falling asleep and easily waking up during the night (Daley et al., 2009). It is estimated that 10% to 15% of the adult population suffers from chronic insomnia disorder, and an additional 25% to 35% have transient or occasional insomnia disorder worldwide (Doghramji, 2006). Patients with insomnia disorder have increased incidences of cardiovascular disease, diabetes, and mental illness compared with healthy controls (Morin and Benca, 2012). Depression is a common complication of insomnia disorder, affecting approximately 50% of insomnia disorder patients (Fortier-Brochu et al., 2012). It is urgently needed to develop effective therapeutic approaches for the management of insomnia disorder.

The gut-central nervous system (CNS) communication plays an important role in the development of sleeping, mood, and mental disorders (Lucas, 2018). Recently, metagenomic sequencing has enabled researchers to reveal the function of the brain-gut microbiota axis in neuropsychiatric disorders. Gut microbiota exhibits a dynamic balance in a healthy state, and a host with dysregulated gut microbiota has increased risks to diseases (Martin et al., 2018). Li et al. have shown that human gut microbiota can regulate the sleep and mental states of the host and that the emotional states also affect the composition of gut microbes, suggesting an important role of the brain-gut microbiota axis in human health (Li et al., 2018). Also, gut microbiota regulates the synthesis of neuroactive molecules, such as acetylcholine, catecholamines, γ-aminobutyric acid, histamine, melatonin, and serotonin (Petra et al., 2015), which may also be influenced by environmental stressors (Huang and Fang, 2020; Sun et al., 2022). For example, gut microbiota modulates the synthesis of neurotransmitter 5-hydroxytryptamine (5-HT) in the CNS through the kynurenine pathway. 5-HT regulates sleep and mood by interacting with 5-HT1A and 5-HT2A autoreceptors, playing important roles in the development of insomnia disorder and depression (Gershon, 2013). These findings suggest that some metabolites may mediate the interaction between the brain and gut microbiota. However, the gut microbiota and serum metabolites involved in insomnia disorder remain largely unknown.

In this study, we employed 16S rDNA sequencing and bioinformatics analysis to investigate the alterations in gut microbiota and serum metabolic profiles as well as their correlations in patients with insomnia disorder. Our results not only provide some bacteria and serum metabolites as potential biomarkers or therapeutic targets for the management of insomnia disorder but also suggest important roles of serum metabolites in mediating the gut microbiota-CNS communication in the pathogenesis of insomnia disorder.

## Materials and Methods

### Participants

A total of 146 patients with primary insomnia disorder (ID) and 48 healthy controls (HC) were recruited from Henan Provincial People’s Hospital (Henan, China) from December 2018 to February 2019. After a strict screening (**Figure S1**), 24 insomnia and 22 healthy samples were included in this study. The inclusion criteria were: 1) aged 18–60 years; 2) insomnia disorder diagnosis was based on the Fifth Edition of the Diagnostic and Statistical Manual of Mental Disorders (Vahia, 2013) and the Chinese Classification of Mental Disorders Version 3 (Chen, 2002); 3) patient with a Pittsburgh Sleep Quality Index (PSQI) ≥ 11. The exclusion criteria were: 1) secondary insomnia disorder resulting from other medical and psychiatric conditions; 2) suicidal ideation and severe mental disorders; 3) past or current alcohol use, smoking, and drug abuse; 4) pregnancy; 5) having taken antibiotics for more than 3 days within 3 months or taking antibiotics during the study; 6) diarrhea within three months of the study or during the study; 7) having taken probiotics and/or prebiotics within 1 year of the study or during the study; 8) a history of mental disorders, neurological diseases, or brain injury; 9) a family history of genetic disorders; 10) a history of major physical illness; 11) total cholesterol > 5.17 mmol/L; 12) fasting glucose > 6.1 mmol/L. This study was approved by the Ethics Committee of Henan Provincial People’s Hospital (No. 2018(59)). All participants provided written informed consents before study initiation.

### Assessment of Sleep Quality, Depression, and Anxiety

Sleep quality was measured by professional neurologists using polysomnography (PSG), a gold standard to measure sleep objectively (Marino et al., 2013), and evaluated using the Insomnia Severity Index (ISI) and PSQI that are widely used in the insomnia field (Morin et al., 2011; Espie et al., 2014). Depression and anxiety were assessed using the Hamilton Depression Rating Scale (HAMD) and the Hamilton Anxiety Rating Scale (HAMA) (Zimmerman et al., 2017), respectively.

### 16s rDNA Amplicon Sequencing

Three fecal samples were collected from each participant during the first bowel movement. To prevent contamination from the environment, fecal samples were collected from the center part of the stool and placed into sterile containers. The samples were then transported on ice, stored at -80°C, and subjected to DNA isolation within 30 min after collection. The 16s rDNA sequencing was performed within 2 weeks after collection. Total DNA was extracted from fecal samples using a QIAamp^®^ Fast DNA stool mini kit (Qiagen, Germany). A multiplexed amplicon library covering the V3–V4 region of the 16S rDNA gene was amplified using the optimized primer sets (forward: 5′-CCTACGGGRSGCAGCAG-3′, reverse: 5′-GGACTACVVGGGTATCTAATC-3′; Sangon Biotech, Shanghai, China) on an Illumina HiSeq2500 PE250 sequencing instrument (Illumina, San Diego, CA, USA). The raw sequence was filtered using Panadaseq software v2.9 (Microsoft Corporation, Redmond, WA, USA), and the clean reads with a length of 220–500 nucleotides were preserved.

### Bioinformatics Analysis

The sequence fragments were assembled in operating taxonomic units (OTUs), taxonomically annotated, and used to assess the structure and membership of the gut microbiota. OTU clustering was conducted as previously described (Edgar et al., 2011; Edgar, 2013). Briefly, the clean reads were sorted with identical sequences according to their abundance. After removing the singletons, the sequences were clustered using Usearch at a similarity of 0.97 and then chimed to obtain OTUs for species classification. The clean reads were compared to the OTU sequences to obtain the final mapped reads. Taxonomic annotation was performed using MEGAN software (http://ab.inf.uni-tuebingen.de/software/megan/) (Huson et al., 2007) to regress the top 100 OTU tables according to the abundance in the taxonomic database of microbial species in NCBI. A comprehensive view of the evolutionary relationships and differences in the abundance of all microorganisms was obtained from the entire taxonomic system.

Alpha rarefaction was analyzed using Faith’s phylogenetic diversity (Faith, 1992), Chao1 (Chao, 1984; Chao and Shen, 2003). Beta diversity was estimated using computing weighted and unweighted UniFrac distance. Principal coordinates analysis (PCoA), redundancy analysis, and heatmap of correlation were plotted using “ggplot2”, “vegan”, and “corrplot” packages of R (version 3.5.1). To identify the biomarkers that differentiate the two groups, the linear-discriminant-analysis-effect-size (LEfSe) method was used under the following conditions: (1) α-value of the factorial Kruskal–Wallis test among the classes < 0.05; (2) the threshold of the logarithmic linear-discriminant-analysis score for the discriminative features > 4.0.

### Ultraperformance Liquid Chromatography-Mass Spectrometry

UPLC-MS was performed to analyze serum metabolites. A total of 5 mL peripheral blood samples were obtained from each participant in the morning after an overnight fast and centrifuged at 3,000 rpm for 10 min to collect the serum samples. Serum metabolites were analyzed using an Agilent 1290 UHPLC hyphenated with an AB Sciex™ 5600 Triple TOF mass spectrometer (Agilent Technologies, Santa Clara, CA, USA) in the ESI negative and positive mode. An Acquity UPLC BEH Amide column (1.7 μm, 2.1 × 10 mm; Waters Corporation, Milford, MA, USA) was used as a stationary phase. The mobile phase consisted of 25 mM ammonium acetate and 25 mM ammonium hydroxide (A) or acetonitrile (B) water solutions. The gradient was as follows: 0–0.5 min, 95% B in A; 0.5–7 min, 95–65% B in A; 7–8 min, 65–40% B in A; 8–9 min, 40% B in A; 9–9.1 min, 40–95% B in A; and 9.1–12 min, 95% B in A. The flow rate was 0.5 mL/min, and the column temperature was 20°C. The mass spectrometer was run in the positive and negative mode using the following parameters: probe heater temperature 650°C, capillary temperature 275°C, spray voltage 4 kV, sheath gas flow 60 Psi, and auxiliary gas flow 60 Psi. Samples were thawed and centrifuged at 13,000 rpm for 10 min before loading. The injection volume was 3 μL. 5% ethanol in phosphate-buffered saline buffer was used as a blank.

### Metabolomics Data Analysis

The raw data from metabolic profiling was filtered, normalized, and standardized by BPI chromatograph as previously described (Smith et al., 2006). The M/S ratio and retention time were used for metabolite identification. The principal component analysis (PCA) and orthogonal-projections-to-latent-structures discriminant analysis (OPLS-DA) were carried out to assess the difference between groups. The concentrations of metabolites were converted to variable importance in the projection (VIP) and fold change for comparison. The metabolites with VIP > 1 were selected (Cho et al., 2008).

Functional annotation analysis was performed using the Kyoto Encyclopedia of Genes and Genomes (KEGG) database. The gene sets were compared with the KEGG gene database using BLAST version 2.2.28+ (http://blast.ncbi.nlm.nih.gov/Blast.cgi) (Kanehisa et al., 2016).

### Statistical Analyses

Data are expressed as the mean ± standard deviation. The results of Alpha diversity analysis were subjected to the Wilcoxon rank-sum test or the Kruskal-Wallis test. The results of beta diversity analysis were subjected to ANOSIM, Adonis, and MRPP analyses. For metabolomics data, the differences between groups were analyzed using the Student *t-*test or Kruskal-Wallis test. Correlations between bacterial genus and serum metabolites were evaluated using the Spearman correlation analysis. A *p*-value < 0.05 was considered statistically significant.

## Results

### Clinical Characteristics of the Participants

The clinical characteristics of the subjects were summarized in **Supplementary Table S1**. No significant differences were observed in gender, body weights, body mass index, heights, and high-density lipoprotein concentrations between patients with insomnia disorder and healthy controls. Compared with healthy controls, patients with insomnia disorder showed significantly higher ISI (1.79 ± 3.07 vs. 19.54 ± 5.99, *p* < 0.001), PSQI (3.21 ± 1.89 vs. 15.87 ± 2.59, *p* < 0.001), HAMD (2.50 ± 2.56 vs. 19.33 ± 8.09, *p <*0.01), HAMA (1.79 ± 2.12 vs. 23.40 ± 11.91, *p* < 0.001), and SHAPS scores. These results suggest that patients with insomnia disorder also have mood disorders.

### Patients With Insomnia Disorder Exhibit Differential Gut Microbiota Compared With Healthy Controls

**Figure 1:**
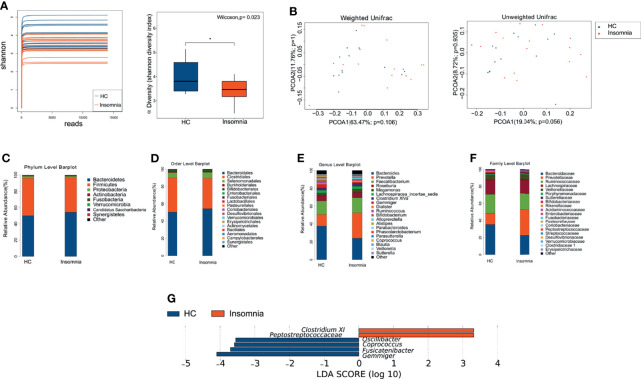
Patients with insomnia showed differential gut microbial profiles compared with healthy controls. **(A)** α-diversity of Shannon index between insomnia and healthy control groups. *p < 0.05. **(B)** Plots of weighted and unweighted UniFrac PCoA of all samples. Orange dots: healthy controls; blue dots: insomnia patients. **(C–F)** Bar graphs of relative abundance of gut bacteria in healthy controls **(A)** and insomnia patients **(B)** at the levels of phylum, order, family, and genus. **(G)** Bar graphs of LEfSe analysis. Orange and blue bars represent the impact degree of enrichment of certain taxa in the healthy controls and insomnia patients, respectively. The LEfSe score threshold was 3 or -3. PCoA, principal coordinates analysis; LEfSe, linear-discriminant-analysis-effect-size; LDA, linear discriminant analysis; HC, Healthy control.

To explore the association of insomnia disorder with gut microbiota, we performed high-throughput sequencing to analyze gut microbial profiles of the fecal samples from the participants. We obtained 406,288 high-quality reads with an average of 34,875 reads per sample. The raw data were assembled in 332 unique OTUs, with 122.5 OTUs per sample. The OTUs were attributed to 9 phyla, 15 classes, 19 orders, 21 families, and 21 genera. Good’s coverage was greater than 99.9% in all samples, indicating a sufficient sequencing depth for the analysis. A significantly reduced Shannon index (p < 0.05) in the ID group indicated that the fecal-microbiota diversity was reduced in patients with insomnia disorder (**Figure 1A**).

On the basis of unweighted and weighted UniFrac distances, the Anosim similarity analysis and MRPP analysis were performed. The positive Anosim (R=0.06 for weighted UniFrac Anosim and R=0.075 for unweighted UniFrac) and MRPP (A= 0.0179 for weighted UniFrac Anosim and A= 0.0093 for unweighted UniFrac) score indicated a larger variation in gut microbial profile between ID and HC group. β-diversity calculated with the unweighted and weighted uniFrac algorithms showed that insomnia disorder patients and healthy controls had no structural difference in gut microbiota (**Figure 1B**).

To further investigate whether insomnia disorder is associated with the alteration in gut microbiota, we analyzed the relative abundance of gut bacteria at the phylum, order, family, and genus levels. As shown in **Figures 1C–F**, the dominant bacterial phyla in both insomnia disorder and healthy control groups were *Bacteroidetes* and *Firmicute*. At the family level, patients with insomnia disorder exhibited significant decreases in family *Bacteroidaceae* and *Ruminococcaceae*, along with a significant increase in family *Prevotellaceae*, compared with healthy controls. At the genus level, patients with insomnia disorder showed a significant decrease in genus *Bacteroides* and a significant increase in genus *Prevotella*, compared with healthy controls. Then, we performed LEfSe analysis to identify potential bacterial biomarkers for insomnia disorder. We found that genus *Gemmige*r and *Fusicatenibacter* were dominant in patients with insomnia disorder, whereas family *Peptostreptococcaceae*, genus *Coprococcus*, genus *Oscillibacter*, and genus *Clostridium XI* were dominant in healthy controls (**Figure 1G**). Taken together, these results suggest that insomnia disorder patients have a different composition of gut microbiota compared with healthy controls. The differentially abundant gut microbes may contribute to the development of insomnia, serving as potential biomarkers and therapeutic targets for the management of insomnia.

### Patients With Insomnia Disorder and Healthy Controls Have Differential Metabolic Profiles

**Figure 2:**
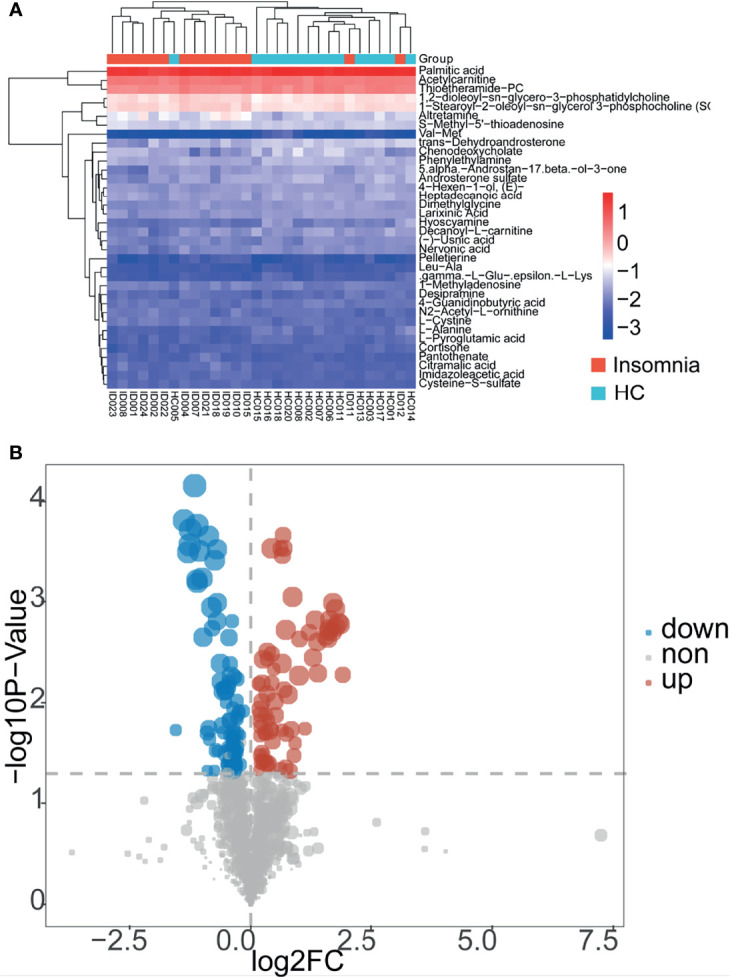
Patients with insomnia showed differential serum metabolic profiles compared with healthy controls. **(A)** A heatmap of metabolites with significantly altered concentrations between insomnia and healthy control groups. Red and blue represent increased and decreased metabolites, respectively, in patients with insomnia compared with those in healthy controls. **(B)** A dot plot of metabolites in the serum samples of the participants. Blue and red dots represent significantly decreased and increased metabolites in the serum samples of insomnia patients compared with those of healthy controls. Gray dots represent nonsignificantly changed metabolites.

**Figure 3:**
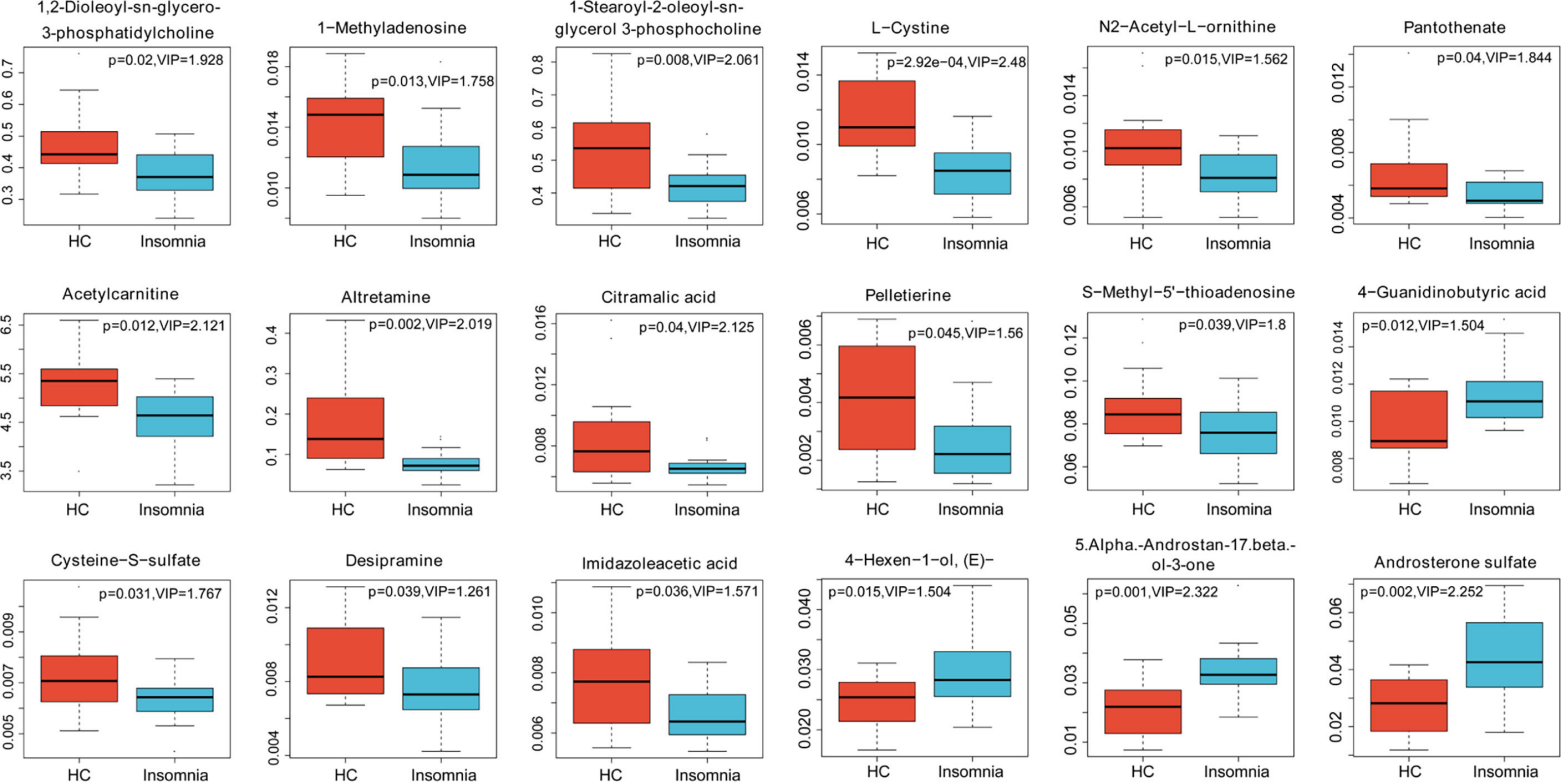
Box plots (part I) of significantly changed metabolites identified in LC-MS analysis. Blue, patients with insomnia; Orange, healthy controls.

**Figure 4:**
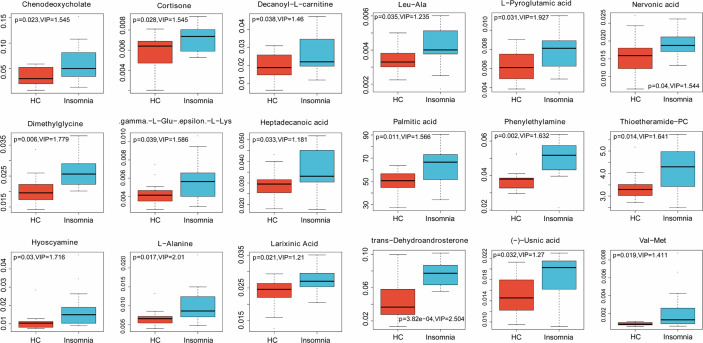
Box plots (part II) of significantly changed metabolites identified in LC-MS analysis. Blue, patients with insomnia; Orange, healthy controls.

**Figure 5:**
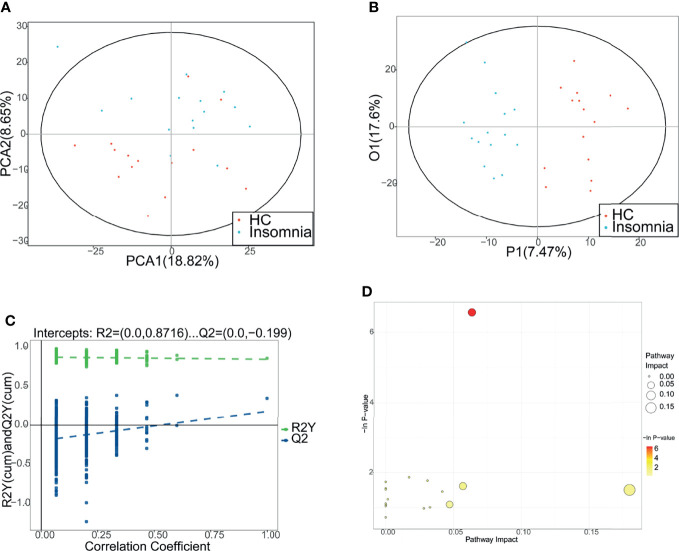
Distinguishment between patients with insomnia and healthy controls using metabolic profiles. **(A, B)** Principal component analysis and orthogona projections to latent structures discriminant analysis (OPLS-DA) were performed to separate patients with insomnia and healthy controls using the metabolic profiles. Red: healthy controls. Blue: patients with insomnia. **(C)** The permutation test of OPLS-DA. **(D)** The metabolic pathway topology analysis. The horizontal axis indicates the −ln(p) values. The vertical axis indicates the impact values. PCA, principal component analysis; P1, 1st predictive principal component; O1, 1st orthogonal principal component.

To explore the association of insomnia disorder with bacterial metabolism, we analyzed the serum metabolic profiles of the subjects using UPLC-MS. As shown in **Figures 2A, B**, **3**, and **4**, compared with healthy controls, patients with insomnia disorder had 97 significantly decreased metabolites, such as androsterone sulfat and chenodeoxycholate, as well as 74 significantly increased metabolites, such as 1,2-dioleoyl-sn-glycero-3-phosphatidylcholine and 1-Methyladenosine, in serum, suggesting that patients with insomnia disorder and healthy controls have differential metabolic profiles. Thus, we sought to distinguish insomnia disorder and healthy control groups using the metabolic data. As shown in **Figures 5A, B**, OPLS-DA provided a better separation of insomnia disorder and healthy control groups than PCA, with an R2 value of 0.876 and a Q2 value of -0.199 that indicate an accurate prediction (**Figure 5C**). These data suggest that differential metabolic profiles can distinguish patients with insomnia disorder from the healthy controls.

Then, we analyzed the metabolic pathways between insomnia disorder and healthy control groups. KEGG enrichment analysis showed that compared with healthy controls, patients with insomnia disorder showed significant changes in 17 metabolic pathways, including 1 upregulated pathway and 16 downregulated pathways (*P* < 0.05 and pathway impact > 0.04; **Table 1** and **Figure 5D**). These data provide possible mechanisms underlying the development of insomnia disorder and suggest that insomnia disorder is associated with gut microbial metabolism.

**Table 1:** Significantly differential metabolic pathways between insomnia disorder patients and healthy controls.

Pathway	Total	Hits	Raw p	- Log (p)	Holm p	FDR	Impact
Pantothenate and CoA biosynthesis	27	1	0.054887	2.9025	1	1	0.18014
Beta-Alanine metabolism	28	1	0.056872	2.8669	1	1	0
Glutathione metabolism	38	1	0.076545	2.5699	1	1	0.0019
Arginine and proline metabolism	77	1	0.15016	1.8961	1	1	0.00447
Cysteine and methionine metabolism	56	4	0.0014079	6.5656	0.11263	0.11263	0.06354
Arginine and proline metabolism	77	2	0.15485	1.8653	1	1	0.01841
Taurine and hypotaurine metabolism	20	1	0.16837	1.7816	1	1	0.03237
Selenoamino acid metabolism	22	1	0.18364	1.6948	1	1	0
Alanine, aspartate and glutamate Metabolism	24	1	0.19863	1.6163	1	1	0.05698
Fatty acid elongation in mitochondria	27	1	0.22063	1.5113	1	1	0
Pantothenate and CoA biosynthesis	27	1	0.22063	1.5113	1	1	0.18014
Beta-Alanine metabolism	28	1	0.22783	1.4791	1	1	0
Steroid hormone biosynthesis	99	2	0.22826	1.4773	1	1	0.04349
Glutathione metabolism	38	1	0.29647	1.2158	1	1	0.0019
Histidine metabolism	44	1	0.33481	1.0942	1	1	0.04715
Phenylalanine metabolism	45	1	0.341	1.0759	1	1	0
Primary bile acid biosynthesis	47	1	0.35322	1.0407	1	1	0.00027
Glycine, serine and threonine Metabolism	48	1	0.35925	1.0237	1	1	0.0342
Fatty acid biosynthesis	49	1	0.36523	1.0072	1	1	0
Fatty acid metabolism	50	1	0.37115	0.99115	1	1	0.02959
Aminoacyl-tRNA biosynthesis	75	1	0.50317	0.68682	1	1	0

### Correlations Between Insomnia-Related Bacterial Genus and Serum Metabolites

**Figure 6:**
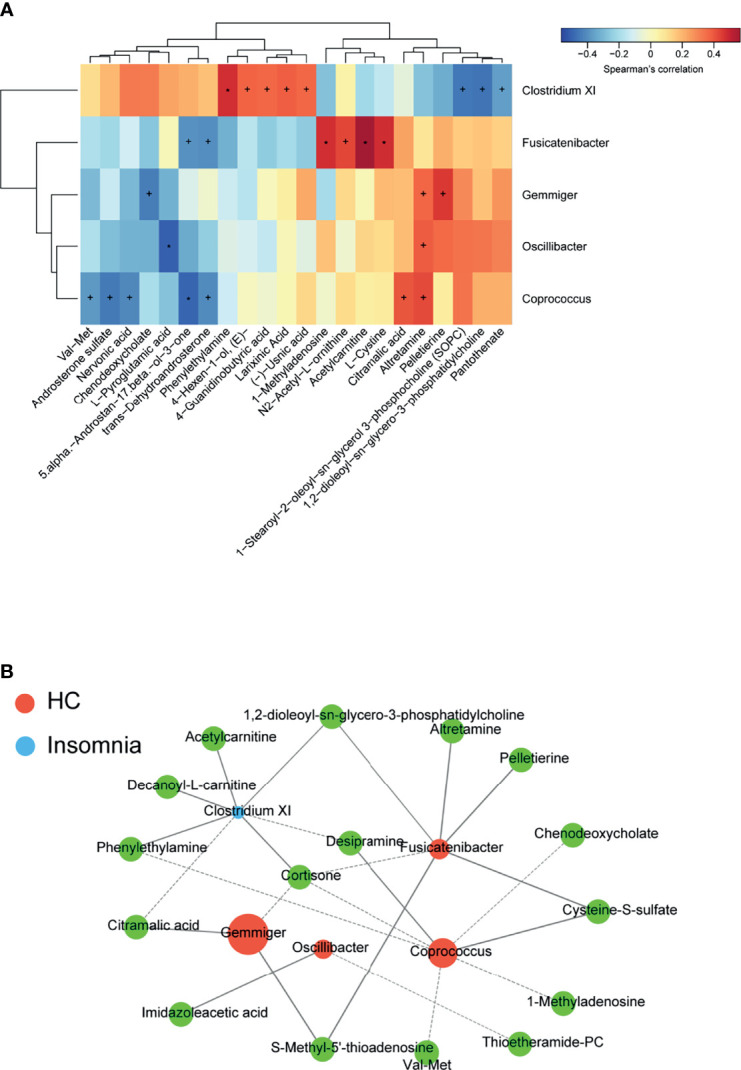
Correlations between insomnia-related gut bacterial genus and serum metabolites. **(A)** A heat map of Spearman’s rank correlation coefficients and p-values of the correlation analysis. ^+^p < 0.05; *p < 0.01. **(B)** A bacteria-metabolite network. Orange dots represent the dominant gut bacteria in insomnia disorder. Blue dots represent the dominant gut bacteria in healthy control. The size of each dot reflects the impact value of each gut bacterial genus. The green dots represent metabolites.

We further performed a Spearman correlation analysis to explore the correlations of the insomnia-related bacterial genus with the serum metabolites. As shown in **Figure 6A** and **Table S2**, genus *Gemmiger* and genus *Fusicatenibacter* that were dominant in insomnia disorder patients were significantly correlated with 3 and 6 metabolites, respectively. Genus *Coprococcus*, *Oscillibacter*, and *Clostridium XI* that were dominant in healthy controls were significantly correlated with 7, 2, and 8 metabolites, respectively. The Spearman r- and *P*-values were summarized in **Table S3**. For example, genus *Fusicatenibacter* positively correlated with antidepressant acetylcarnitine (*r* = 0.5651, *P* = 0.0011); genus *Clostridium XI* positively correlated with mood regulator phenylethylamine (*r* = 0.4755, *P* = 0.0079); genus *Clostridium XI* negatively correlated with potential insomnia alleviator 3-phosphocholine (*r* = -0.4503, *P* = 0.0125). **Figure 6B** illustrates a bacteria-metabolite network. These data suggest that alterations in gut microbiota may affect the production or degradation of sleep-related metabolites, mediating the gut microbiota-CNS communication in insomnia disorder.

### Correlations Between Gut Microbiota and Sleep Score as Well as Metabolites and Sleep Score

**Figure 7:**
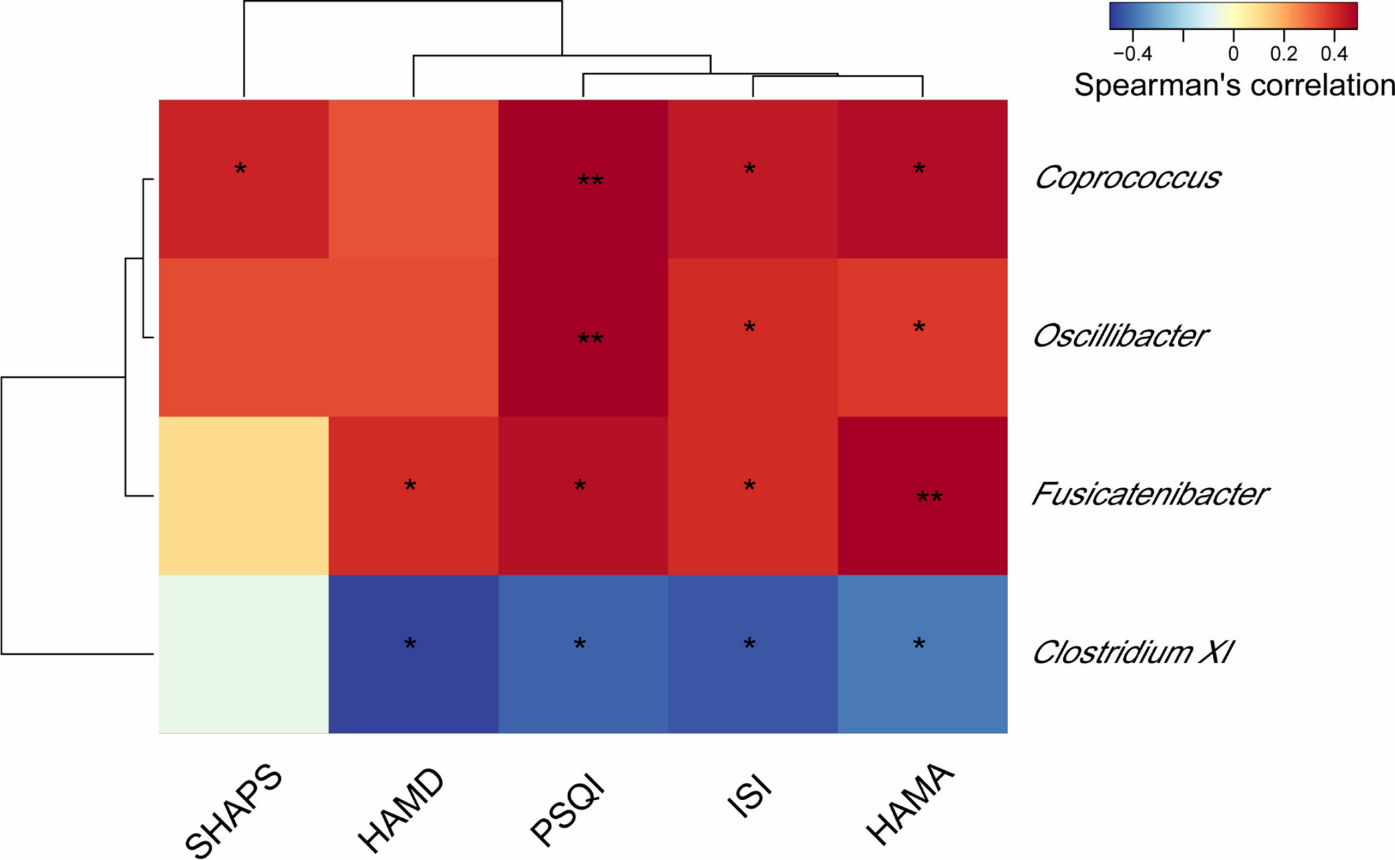
Heat map of the Spearman correlation analysis between gut microbes and sleep/mood index. *P < 0.05; **P < 0.01. PSQI, Pittsburgh Sleep Quality Index; ISI, Insomnia Severity Index; HAMA, Hamilton Anxiety Rating Scale; HAMD, Hamilton Depression Rating Scale; SHAPS, Snaith-Hamilton Pleasure Scale.

**Figure 8:**
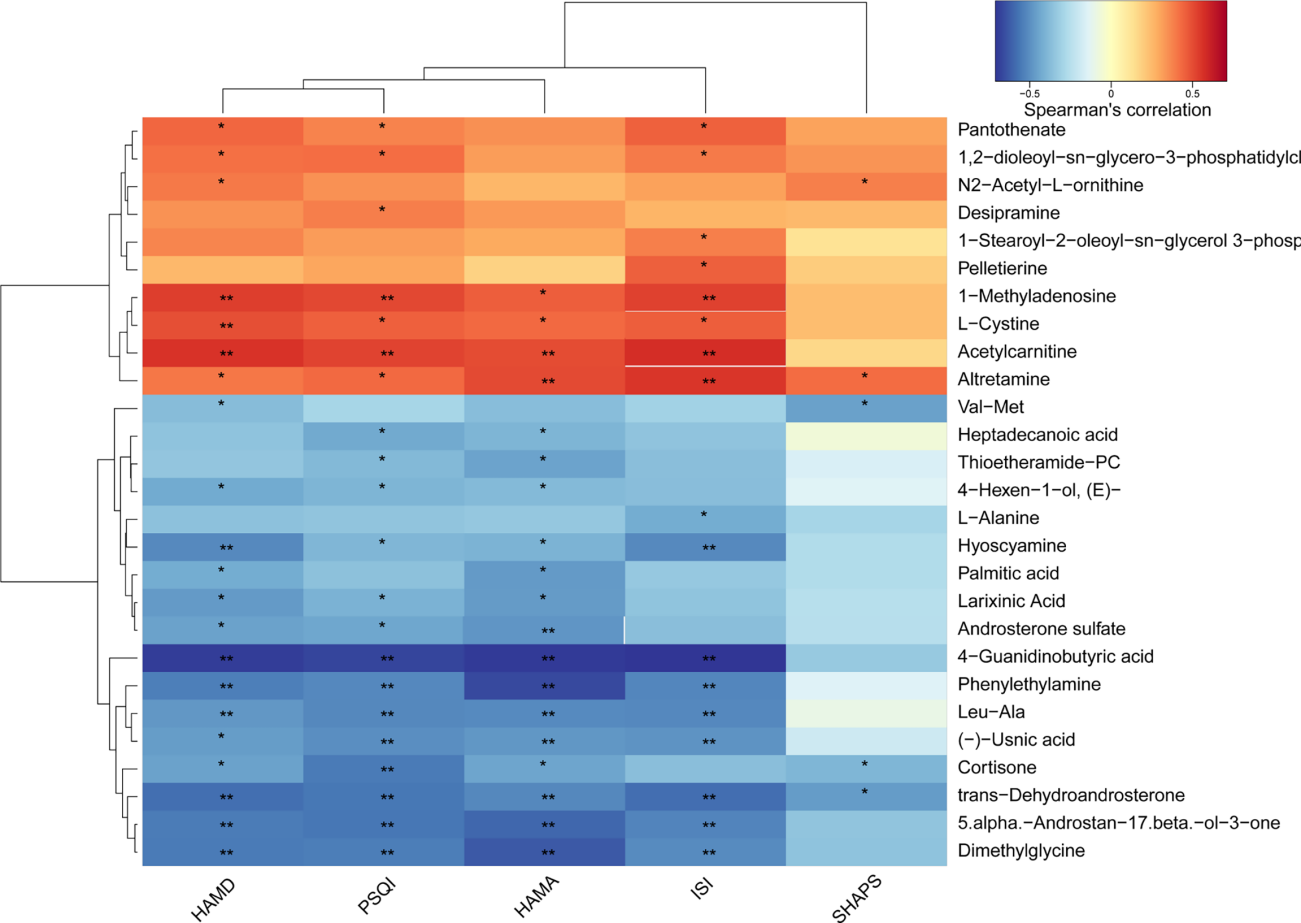
Heat map of the Spearman correlation analysis between serum metabolites and sleep/mood index. *P < 0.05; **P < 0.01. PSQI, Pittsburgh Sleep Quality Index; ISI, Insomnia Severity Index; HAMA, Hamilton Anxiety Rating Scale; HAMD, Hamilton Depression Rating Scale; SHAPS, Snaith-Hamilton Pleasure Scale.

To investigate whether the changes in the gut microbiota and serum metabolite profile are associated with the severity of insomnia, we assessed the correlations between gut microbiota and sleep/mood indexes as well as metabolites and sleep/mood indexes. As shown in **Figure 7**, the abundance of genus *Coprococcus*, *Oscillibacter*, and *Fusicatenibacter* significantly, positively correlated with at least 3 sleep/mood indexes whereas genus *Clostridium XI* negatively correlated with ISI, PSQI, HAMA, and HAMD (all *P* < 0.05). We also observed significant correlations between serum metabolites and sleep/mood indexes (**Figure 8**). For example, acetylcarnitine and 3-phosphocholine serum levels positively correlated with ISI, PSQI, HAMA, and HAMD (all *P* < 0.05, except for 3-phosphocholine with HAMA) whereas phenylethylamine serum level negatively correlated with these indexes (all *P* < 0.05). These results suggest that alterations in gut microbiota and serum metabolites are related to sleep quality.

## Discussion

In this study, using 16s rDNA sequencing and serum metabolic profiling, we identified dominant fecal bacteria and significantly changed serum metabolites in patients with insomnia compared with healthy controls. Pearson’s correlation analysis revealed significant correlations among the altered gut bacteria and serum metabolites. Our results not only provide some fecal bacteria and serum metabolites as potential biomarkers or therapeutic targets for insomnia disorder but also highlight the interactions between gut bacteria and serum metabolites that may play important roles in the pathogenesis of insomnia disorder.

In this study, we observed a remarkable change in the composition of gut microbiota in patients with insomnia disorder compared with healthy controls. At the family level, *Bacteroidaceae* and *Ruminococcaceae* were significantly decreased, whereas *Prevotellaceae* was significantly increased in insomnia disorder patients. Genus *Gemmiger* and *Fusicatenibacter* were dominant bacterial genus in patients with insomnia disorder. Studies have shown that imbalanced gut microbiota induces alterations in circulating bacterial products, leading to sleeping disorders by affecting the neural, endocrine, and immune systems (Krueger et al., 1982; Reynolds et al., 2017). For example, exposure to antibiotics, such as neomycin and metronidazole, suppresses the synthesis of sleep-inducing substance factor S in mice due to a remarkable reduction in gut bacteria, leading to a decrease in slow-wave sleep and an increase in sleep latency (Brown et al., 1990). Similarly, minocycline administration reduces slow-wave sleep for up to three consecutive nights in healthy male students (Nonaka et al., 1983). Hermann et al. have reported that *Salmonella abortus* endotoxin induces the release of cytokines and neurohormones in humans, thereby reducing the duration of non-rapid eye movement sleep while increasing waking and sleep latency (Pollmacher et al., 1993).

Increased levels of proinflammatory cytokines contribute to the development of sleep disturbances (Park and Chung, 2016). On the other hand, sleep disturbance is associated with inflammatory disease risk (Irwin et al., 2016). The altered gut bacteria identified in our study have been closely linked to the production of inflammatory cytokines. Family *Prevotellaceae* is considered a primary driver of intestinal inflammation by facilitating TNF-α and IL-6 production and activating Th17 and B cells in the intestinal microenvironment (Grigg and Sonnenberg, 2017). Increasing the abundance of genus *Prevotella* and the like promotes the onset of various immune diseases (Larsen, 2017). On the contrary, the reduction of family *Ruminococcaceae* occurs in diabetes and other inflammatory diseases and is associated with the production of inflammatory cytokines *via* inducing colonic epithelium barrier disruption (Poroyko et al., 2016). In gut microbiota of insomnia patients, we observed significant increases in the abundance of family *Prevotellaceae* and genus *Prevotella* as well as a significant reduction in family *Ruminococcaceae* compared with those in healthy controls. These alterations may increase the risk of developing immune diseases in insomnia patients. Genus *Fusicatenibacter* produces anti-inflammatory short-chain fatty acid butyrate (Slingerland et al., 2017), and the abundance of genus *Fusicatenibacter* negatively correlates with the concentrations of proinflammatory cytokines in human serum, such as IL-6, TNF-α, and IL-1β (Voorhies et al., 2019). Although genus *Fusicatenibacter* was dominant in patients with insomnia disorder, the beneficial effect of genus *Fusicatenibacter* on the immune system may be overridden by proinflammatory bacteria. Also, gut microbiota produces neurotransmitters and their analogs. Gamma-aminobutyric acid (GABA) is an antianxiety hormone that facilitates relaxation and sleep (Gottesmann, 2002). The metabolite of genus *Oscillibacter* shares a similar chemical structure and properties with GABA and binds to the GABA receptor in the CNS (Leclercq et al., 2014). This may explain why patients with sleep disorders have decreased genus *Oscillibacter* (Li et al., 2017).

Depression and anxiety are two common complications in patients with insomnia disorder (Swanson et al., 2011). Indeed, our results showed that patients with insomnia disorder exhibited remarkably higher HAMD and HAMA scores than healthy controls, suggesting that patients with insomnia disorder also have mood disorders. Studies have suggested that alteration in gut microbiota is responsible for the occurrence of depression (Zheng et al., 2016). Thus, we sought to explore the correlation between gut microbiota and serum metabolic profiles in insomnia disorder patients. As a promising antidepressant by evoking epigenetic exchange in the genes related to glucose metabolism (Nasca et al., 2013), acetylcarnitine also regulates sleep rhythm and quality. Decreased acetylcarnitine level correlates with increased frequency of fragmented wakefulness and rapid eye movement sleep (Tafti et al., 2003; Miyagawa et al., 2013). Our results showed for the first time that acetylcarnitine serum level positively correlated with genus *Fusicatenibacter* in the human gut. Both acetylcarnitine and genus *Fusicatenibacter* possess anti-inflammatory properties (Zanelli et al., 2005; Slingerland et al., 2017; Voorhies et al., 2019). Therefore, increases in gut *Fusicatenibacter* and serum acetylcarnitine may reduce the risk of developing mood disorders and immune diseases in insomnia patients. However, the underlying mechanism of the correlation between *Fusicatenibacter* and acetylcarnitine needs further investigation. Phenylethylamine is a bacteria-synthesized mood regulator that can enter the CNS owing to its high lipophilicity. Phenylethylamine not only improves sleep quality but also enhances mood and prevents depression by participating in neurotransmitter formation (Mermigkis et al., 2013). Tavakkol et al. have revealed the production of phenylethylamine by genus *Clostridium*, consistent with our finding showing that phenylethylamine serum level positively correlated with gut *Clostridium XI* (Tavakkol et al., 1985). Phosphatidylcholine, also named 3-phosphocholine, has been shown to alleviate psychiatric diseases and insomnia (Rao et al., 2015), negatively correlating with gut genus *Clostridium XI* in our study possibly because genus *Clostridium* can degrade phosphatidylcholine (Nagahama et al., 2007).

This study has some limitations. First, the sample size is relatively small. Future studies with larger sample sizes and more complex cases are needed. Second, there were age differences between groups. Matching for age and gender will be conducted in future studies.

## Conclusions

In summary, we demonstrated that patients with insomnia disorder had significantly differential composition of gut microbiota and serum metabolic profiles compared with healthy controls. The altered bacteria genus was closely related to serum metabolites involved in immunoregulation and amino acid or lipid metabolism. These findings provide new information about the pathogenesis of insomnia disorder and suggest that restoring healthy gut microbiota is a promising therapeutic strategy for the treatment of insomnia.

## Data Availability Statement

The original contributions presented in the study are included in the article/**Supplementary Material**. Further inquiries can be directed to the corresponding author.

## Ethics Statement

The studies involving human participants were reviewed and approved by the Ethics Committee of Henan Provincial People’s Hospital, Ethics No. 2018(59). The patients/participants provided their written informed consent to participate in this study.

## Author Contributions

Conceptualization, JZ and YL. Methodology, XW. Software, ZL. Validation, ZZ, SD and GL. Formal Analysis, XW. Investigation, BC. Resources, ZZ. Data Curation, FY. Writing – Original Draft Preparation, JZ. Writing – Review and Editing, JZ. Visualization, ZL. Supervision, YL. Project Administration, JZ. Funding Acquisition, YL.

## Funding

This research was funded by the National Key R&D Program of China [grant number 2017YFB1002502]; Henan Province Young and Middle-aged Health Science and Technology Innovation Talents Training Project - Leading Talents [grant number YXKC2020004]; and Science and Technology Project of Science and Technology Department of Henan Province [grant number 212102310737]; Key R&D and Promotion Project of Henan Province (Science and Technology research) [grant number 222102310198].

## Conflict of Interest

The authors declare that the research was conducted in the absence of any commercial or financial relationships that could be construed as a potential conflict of interest.

## Publisher’s Note

All claims expressed in this article are solely those of the authors and do not necessarily represent those of their affiliated organizations, or those of the publisher, the editors and the reviewers. Any product that may be evaluated in this article, or claim that may be made by its manufacturer, is not guaranteed or endorsed by the publisher.

## Supplementary Material

Supplementary Figure 1 A schematic overview of recruitment of participants. ID, insomnia disorder.

## Abbreviations

CNS, central nervous system; 5-HT, 5-hydroxytryptamine; ID, insomnia disorder; HC, healthy controls; PSQI, Pittsburgh Sleep Quality Index; PSG, polysomnography; ISI, Insomnia Severity Index; HAMD, Hamilton Depression Rating Scale; HAMA, Hamilton Anxiety Rating Scale; OTUs, operating taxonomic units; PCoA, Principal coordinates analysis; LEfSe, linear-discriminant-analysis-effect-size; UPLC-MS, Ultraperformance liquid chromatography-mass spectrometry; PCA, principal component analysis; OPLS-DA, orthogonal-projections-to-latent-structures discriminant analysis; VIP, variable importance in the projection; KEGG, Kyoto Encyclopedia of Genes and Genomes.
