# Supplementary figures and images for: Alterations in Gut Microbiota Are Correlated With Serum Metabolites in Patients With Insomnia Disorder
Source: Front Cell Infect Microbiol. 2022 Feb 17;12:722662. doi: 10.3389/fcimb.2022.722662 (PMC8892143; doi:10.3389/fcimb.2022.722662)

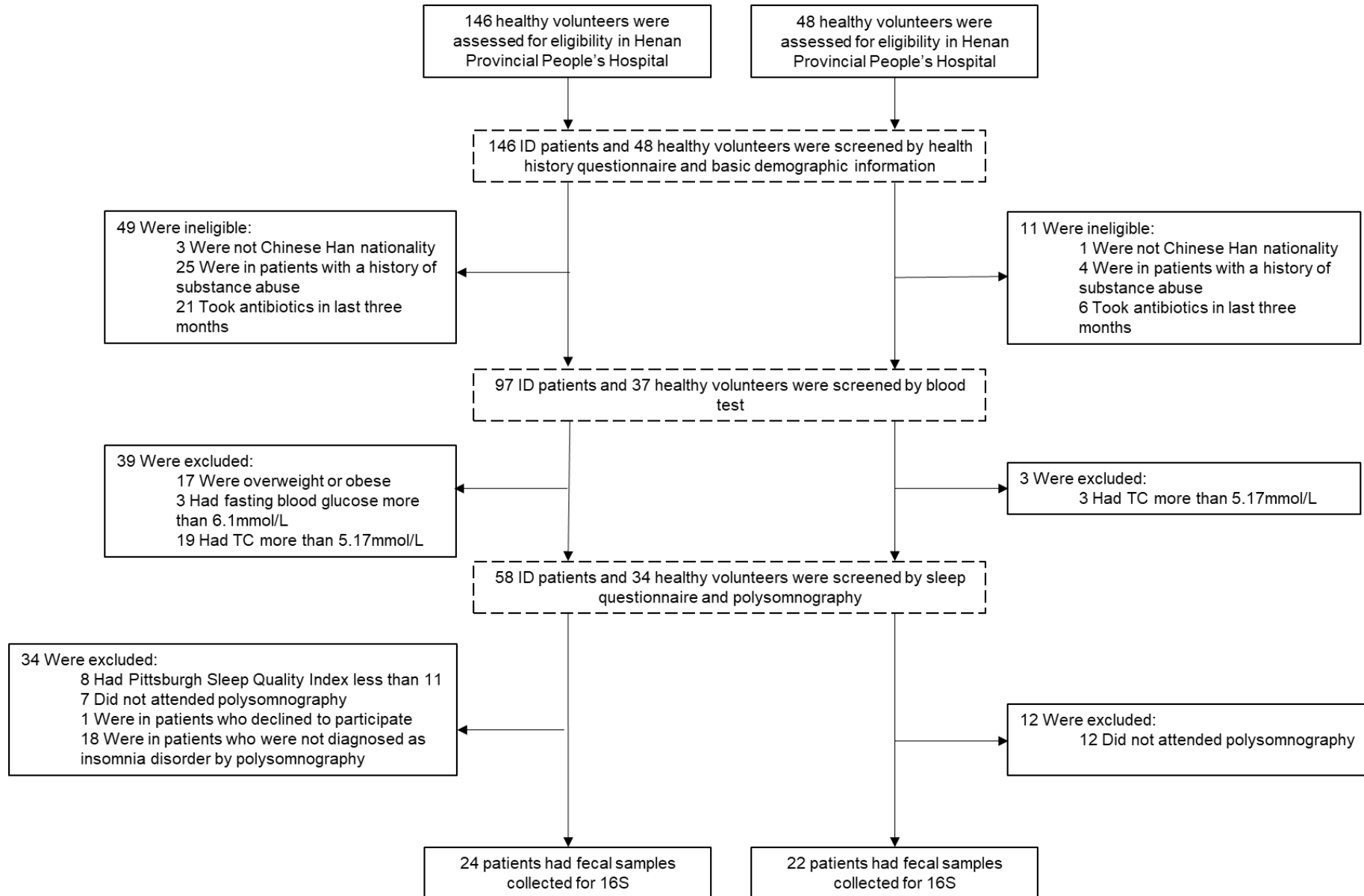

**Supplementary Figure S1.** Participants recruitment flow chart

Supplement: Supplementary Figure 1 — A schematic overview of recruitment of participants. ID, insomnia disorder. [file Image_1.pdf]
