# Supplementary material for: Alterations in Gut Microbiota Are Correlated With Serum Metabolites in Patients With Insomnia Disorder
Source: Front Cell Infect Microbiol. 2022 Feb 17;12:722662. doi: 10.3389/fcimb.2022.722662 (PMC8892143; doi:10.3389/fcimb.2022.722662)
Supplement: Supplementary file 2 [file Table_1.docx]

**Supplementary Table S1.** Clinical characteristics of the participants

| **Characteristics** | **Healthy controls (n=22)** | **Patients with insomnia disorder a (n=24)** | ***t(X^2^)* value** | ***P* value** |
| --- | --- | --- | --- | --- |
| Age | 29.93±7.98 | 49.00±12.12 | -3.55 | <0.01 |
| Gender [n (%)] |  |  | 1.43 | 0.232 |
| Male | 6 (40.0%) | 3 (20.0%) |  |  |
| Female | 9 (60.0%) | 12 (80.0%) |  |  |
| Weight (kg) | 62.54±12.66 | 60.93±8.07 | -0.12 | 0.908 |
| Height (cm) | 169.85±0.89 | 162.13±0.82 | -1.92 | 0.055 |
| Body mass index (kg/m^2^) | 21.77±2.95 | 23.13±2.02 | -1.43 | 0.153 |
| Total cholesterol (mmol/L) | 4.35±0.73 | 5.20±0.84 | -2.31 | 0.021 |
| HDL (mmol/L) | 1.44±0.30 | 1.46±0.32 | -0.29 | 0.775 |
| PSQI | 3.21±1.89 | 15.87±2.59 | -4.63 | <0.01 |
| ISI | 1.79±3.07 | 19.54±5.99 | -4.48 | <0.01 |
| HAMA | 1.79±2.12 | 23.40±11.91 | -4.61 | <0.01 |
| HAMD | 2.50±2.56 | 19.33±8.09 | -4.38 | <0.01 |
| SHPAS | 23.00±4.67 | 29.38±9.00 | -2.20 | 0.028 |

HDL, high-density lipoprotein; PSQI, Pittsburgh sleep quality index; ISI, insomnia severity index; HAMA, Hamilton anxiety rating scale; HAMD, Hamilton depression rating scale; SHAPS, Snaith-Hamilton pleasure scale.
