# Supplementary material for: Alterations in Gut Microbiota Are Correlated With Serum Metabolites in Patients With Insomnia Disorder
Source: Front Cell Infect Microbiol. 2022 Feb 17;12:722662. doi: 10.3389/fcimb.2022.722662 (PMC8892143; doi:10.3389/fcimb.2022.722662)
Supplement: Supplementary file 3 [file Table_2.docx]

**Supplementary Table S2.** Correlations between insomnia-related bacterial genus and serum metabolites

| **Genus** | **Metabolites** | **r value** | **P value** | **number** | **Metabolites** | **r value** | **P value** | **number** |
| --- | --- | --- | --- | --- | --- | --- | --- | --- |
| g_Clostridium XI | Phenylethylamine | 0.47548 | 0.0494 | 5 | 1-Stearoyl-2-oleoyl-sn-glycerol 3-phosphocholine (SOPC) | 0.450291 | 0.012527 | 3 |
|  | 4-Hexen-1-ol | 0.373162 | 0.0476 |  | 1,2-dioleoyl-sn-glycero-3-phosphatidylcholine | 0.45943 | 0.010647 |  |
|  | 4-Guanidinobutyric acid | 0.364468 | 0.047686 |  | Pantothenate | 0.364691 | 0.04754 |  |
|  | Larixinic acid | 0.384976 | 0.035664 |  |  |  |  |  |
|  | (-)-Usnic acid | 0.364468 | 0.035664 |  |  |  |  |  |
| g_Fusicatenibacter | 1-Methyladenosine | 0.475342 | 0.007939 | 4 | 5.alpha.-Androstan-17.beta.-ol-3-one | 0.39007 | 0.033093 | 2 |
|  | N2-Acetyl-L-ornithine | 0.431927 | 0.007939 |  | trans-Dehydroandrosterone | 0.399867 | 0.028571 |  |
|  | Acetylcarnitine | 0.565068 | 0.00114 |  |  |  |  |  |
|  | L-Cystine | 0.478905 | 0.00742 |  |  |  |  |  |
| g_Gemmiger | Altretamine | 0.369241 | 0.044635 | 2 | Chenodeoxycholate | 0.437347 | 0.015658 | 1 |
|  | Pelletierine | 0.456043 | 0.011314 |  |  |  |  |  |
| g_Oscillibacter | Altretamine | 0.391319 | 0.032486 | 1 | L-Pyroglutamic acid | 0.505509 | 0.004378 | 1 |
| g__Coprococcus | Citramalic acid | 0.401205 | 0.027994 | 2 | Val-Met | 0.374444 | 0.041489 | 3 |
|  | Altretamine | 0.423061 | 0.019842 |  | Androsterone sulfate | 0.444694 | 0.01381 |  |
|  |  |  |  |  | Nervonic acid | 0.412356 | 0.0393 |  |
