# Supplementary material for: Alterations in Gut Microbiota Are Correlated With Serum Metabolites in Patients With Insomnia Disorder
Source: Front Cell Infect Microbiol. 2022 Feb 17;12:722662. doi: 10.3389/fcimb.2022.722662 (PMC8892143; doi:10.3389/fcimb.2022.722662)
Supplement: Supplementary file 4 [file Table_3.docx]

**Supplementary Table S3.** Spearman correlation between gut microbiome genus and metabolites.

|  | *Gemmiger* | | *Coprococcus* | | *Fusicatenibacter* | | *Oscillibacter* | | *Clostridium XI* | |
| --- | --- | --- | --- | --- | --- | --- | --- | --- | --- | --- |
|  | r | p | r | p | r | p | r | p | r | p |
| 4-Guanidinobutyric acid | -0.0024 | 0.9898 | -0.0361 | 0.8497 | -0.2476 | 0.1871 | -0.1465 | 0.4399 | 0.3645 | 0.0477 |
| 4-Hexen-1-ol, (E)- | -0.1680 | 0.3747 | -0.0089 | 0.9627 | -0.1603 | 0.3974 | -0.1086 | 0.5677 | 0.3732 | 0.0422 |
| 5.alpha.-Androstan-17.beta.-ol-3-one | -0.0950 | 0.6174 | -0.4926 | 0.0057 | -0.3901 | 0.0331 | -0.3361 | 0.0694 | 0.2140 | 0.2562 |
| Androsterone sulfate | -0.1373 | 0.4693 | -0.4447 | 0.0138 | -0.2135 | 0.2573 | -0.2838 | 0.1285 | 0.1939 | 0.3045 |
| Chenodeoxycholate | -0.4373 | 0.0157 | -0.1947 | 0.3026 | -0.2652 | 0.1567 | -0.3179 | 0.0869 | 0.3219 | 0.0828 |
| Larixinic Acid | 0.0568 | 0.7658 | 0.0230 | 0.9041 | -0.2035 | 0.2808 | 0.0120 | 0.9497 | 0.3850 | 0.0357 |
| L-Pyroglutamic acid | -0.3176 | 0.0872 | -0.2712 | 0.1472 | 0.0062 | 0.9739 | -0.5055 | 0.0044 | 0.2361 | 0.2092 |
| Nervonic acid | -0.2980 | 0.1097 | -0.4124 | 0.0235 | -0.1216 | 0.5222 | -0.3156 | 0.0893 | 0.3237 | 0.0810 |
| Phenylethylamine | -0.1302 | 0.4929 | -0.1269 | 0.5040 | -0.3168 | 0.0880 | -0.0875 | 0.6458 | 0.4755 | 0.0079 |
| trans-Dehydroandrosterone | -0.0345 | 0.8564 | -0.4072 | 0.0255 | -0.3999 | 0.0286 | -0.2455 | 0.1910 | 0.1752 | 0.3544 |
| (-)-Usnic acid | 0.2241 | 0.2338 | 0.1236 | 0.5154 | -0.2518 | 0.1795 | -0.0832 | 0.6618 | 0.3645 | 0.0477 |
| Val-Met | -0.2936 | 0.1154 | -0.3744 | 0.0415 | -0.1839 | 0.3306 | -0.1863 | 0.3242 | 0.1063 | 0.5760 |
| 1,2-dioleoyl-sn-glycero-3-phosphatidylcholine | 0.1712 | 0.3658 | 0.2090 | 0.2678 | 0.1645 | 0.3849 | 0.3370 | 0.0686 | -0.4594 | 0.0106 |
| 1-Methyladenosine | -0.2023 | 0.2836 | -0.0292 | 0.8782 | 0.4753 | 0.0079 | 0.1701 | 0.3689 | -0.2733 | 0.1439 |
| 1-Stearoyl-2-oleoyl-sn-glycerol 3-phosphocholine | 0.2900 | 0.1201 | 0.3408 | 0.0654 | 0.1514 | 0.4245 | 0.3450 | 0.0619 | -0.4503 | 0.0125 |
| Acetylcarnitine | 0.0158 | 0.9339 | 0.0578 | 0.7618 | 0.5651 | 0.0011 | 0.1287 | 0.4980 | -0.2109 | 0.2633 |
| Altretamine | 0.3692 | 0.0446 | 0.4231 | 0.0198 | 0.0862 | 0.6507 | 0.3913 | 0.0325 | -0.2720 | 0.1460 |
| Citramalic acid | 0.1805 | 0.3398 | 0.4012 | 0.0280 | 0.2362 | 0.2088 | 0.1823 | 0.3349 | -0.0671 | 0.7246 |
| L-Cystine | 0.1867 | 0.3231 | 0.0669 | 0.7254 | 0.4789 | 0.0074 | 0.1120 | 0.5558 | -0.1500 | 0.4288 |
| N2-Acetyl-L-ornithine | 0.1195 | 0.5293 | 0.1128 | 0.5527 | 0.4319 | 0.0171 | -0.0363 | 0.8490 | 0.0448 | 0.8141 |
| Pantothenate | 0.2677 | 0.1526 | 0.2103 | 0.2646 | 0.1380 | 0.4670 | 0.3050 | 0.1013 | -0.3647 | 0.0475 |
| Pelletierine | 0.4560 | 0.0113 | 0.0642 | 0.7360 | 0.2088 | 0.2681 | 0.3606 | 0.0503 | -0.3266 | 0.0782 |
